# Adipose Tissue-Derived Human Serum Amyloid A Does Not Affect Atherosclerotic Lesion Area in hSAA1^+/−/^ApoE^−/−^ Mice

**DOI:** 10.1371/journal.pone.0095468

**Published:** 2014-04-21

**Authors:** Sofie Ahlin, Maja Olsson, Anna S. Wilhelmson, Kristina Skålén, Jan Borén, Lena M. S. Carlsson, Per-Arne Svensson, Kajsa Sjöholm

**Affiliations:** 1 Department of Molecular and Clinical Medicine, Institute of Medicine, The Sahlgrenska Academy at the University of Gothenburg, Gothenburg, Sweden; 2 Department of Internal Medicine and Clinical Nutrition, Institute of Medicine, The Sahlgrenska Academy at the University of Gothenburg, Gothenburg, Sweden; University of Catanzaro Magna Graecia, Italy

## Abstract

Chronically elevated serum levels of serum amyloid A (SAA) are linked to increased risk of cardiovascular disease. However, whether SAA is directly involved in atherosclerosis development is still not known. The aim of this study was to investigate the effects of adipose tissue-derived human SAA on atherosclerosis in mice. hSAA1^+/−^ transgenic mice (hSAA1 mice) with a specific expression of human SAA1 in adipose tissue were bred with ApoE-deficient mice. The hSAA1 mice and their wild type (wt) littermates were fed normal chow for 35 weeks. At the end of the experiment, the mice were euthanized and blood, gonadal adipose tissue and aortas were collected. Plasma levels of SAA, cholesterol and triglycerides were measured. Atherosclerotic lesion areas were analyzed in the aortic arch, the thoracic aorta and the abdominal aorta in *en face* preparations of aorta stained with Sudan IV. The human SAA protein was present in plasma from hSAA1 mice but undetectable in wt mice. Similar plasma levels of cholesterol and triglycerides were observed in hSAA1 mice and their wt controls. There were no differences in atherosclerotic lesion areas in any sections of the aorta in hSAA1 mice compared to wt mice. In conclusion, our data suggest that adipose tissue-derived human SAA does not influence atherosclerosis development in mice.

## Introduction

Atherosclerosis is considered to be an inflammatory condition [1]. Patients with atherosclerosis display moderately elevated levels of clinical markers for inflammation, including C-reactive protein and serum amyloid A (SAA) [2], [3]. SAA is suggested as a predictor for cardiovascular disease [2]–[5] and the SAA protein is also present in the atherosclerotic lesion [6]–[8]. However, whether SAA directly influences the development of atherosclerosis is unclear.

SAA1 and SAA2 are the acute phase isoforms of the serum amyloid A protein family. In the acute phase, SAA is produced by the liver [9], [10] and serum levels can rise 1000-fold in response to inflammatory stimuli [11], [12]. However, the adipocyte is the main source of SAA during non-acute phase in humans, and obese individuals chronically display moderately elevated levels of SAA [13], [14]. SAA has been ascribed many different functions of which some could influence the development of atherosclerosis [15]–[22]. In the circulation, SAA is an apolipoprotein and associates with the high density lipoprotein (HDL) particle [23]. It has been suggested that SAA is pro-atherogenic, for example by impairing reverse cholesterol transport [15] or by promoting lipoprotein retention in the vessel wall [8], [19], [20], [24]. However, data suggesting anti-atherogenic functions of SAA have also been presented [18], [22], [25]–[27]. Furthermore, when studying direct effects of SAA or SAA peptides on atherosclerosis *in vivo*, results are conflicting, both increase and decrease in atherosclerosis development have been reported [26], [28]. Hence, whether SAA actively influences the development of atherosclerotic lesions needs to be further investigated.

We have previously reported the establishment of a transgenic mouse strain expressing human SAA1 in the adipose tissue [29]. The mouse model mimics the state of non-acute phase in humans where SAA originates from adipose tissue. As in humans, the SAA protein associates with the HDL-particle in the hSAA1^+/−^ transgenic mice (hSAA1 mice) [29]. Hence, our mouse model gives us an opportunity to investigate the effects of adipose tissue-derived human SAA on atherosclerosis *in vivo*.

## Materials and Methods

### Ethics Statement

The protocol for this study was approved by the local Ethics Committee for Animal Studies at the Administrative Court of Appeals (Gothenburg, Sweden) (Permit numbers 281-2008, 328-2009, 264-2012).

### Animals

We have previously reported the generation of the hSAA1^+/−^ transgenic mice expressing human SAA1 under the control of the aP2 promoter in the adipose tissue [29]. To obtain hSAA1 mice that spontaneously develop atherosclerosis, female hSAA^+/−^ mice were mated with male ApoE^−/−^ mice, then back-crossed for 3 generations with ApoE^−/−^ mice to obtain hSAA1^+/−^ mice and wild type (wt) littermates on a homozygous ApoE-deficient background. Only male animals were used in the current experiments. The animals were weaned at 3 weeks of age and housed 3–5 per cage with free access to food and water. They were kept in a 12-hour dark-light cycle under permanent temperature conditions (25°C). Body weight was recorded weekly from 11 weeks of age until at the end of the experiment. At 35 weeks of age, the animals were fasted for 4 hours and euthanized under Isoba Vet (Schering-Plough, UK) anesthesia. Blood was collected with heart puncture before perfusion of the circulatory system with phosphate buffered saline. The aortic arch and the descending part of the aorta were dissected and placed in paraformaldehyde for subsequent *en face* preparation. Gonadal adipose tissue was excised, snap frozen in liquid nitrogen and stored at −80°C for further analysis.

### En Face Preparations of Aorta and Quantification of Atherosclerotic Lesions

The aortas were dissected free from perivascular tissue, cut open longitudinally and pinned out flat on black silicone coated plates. The atherosclerotic lesions were stained with Sudan IV (Sigma-Aldrich, St. Louis, MO) and digital images were captured. Computer-assisted quantification of atherosclerotic lesion area was performed with BioPix IQ 2.2.1 (Gothenburg, Sweden). The extent of the atherosclerotic lesions was calculated as the percentage of the aortic surface covered by atherosclerotic lesions.

### RNA Preparations and Gene Expression Analysis

Tissue Lyser (Qiagen, Chatsworth, CA) was used to homogenize gonadal adipose tissue before subsequent RNA isolation with the RNeasy Lipid Tissue Mini kit (Qiagen). cDNA was generated from the RNA preparations using the high capacity cDNA Reverse Transcription kit (Applied Biosystems, Foster City, CA). Gene expression was assessed using multiplex real-time PCR according to standard protocol using standard curve quantification. The following TaqMan Gene expression assays were used: rplp0 (Mm99999273_gh), SAA1/2 (Hs00761940_s1), Saa3 (Mm00441203_m1). Amplification and detection of PCR-products were performed using ViiA7 real-time PCR systems (Applied Biosystems) and data was analyzed with ViiA7 ROU software (Applied Biosystems).

### Plasma Analyses

Plasma levels of human and mouse SAA were assessed using the human SAA ELISA kit (Biosource, Camarillo, CA) and the mouse SAA ELISA kit (Tridelta Development Ltd, Kildare, Ireland), respectively. Plasma levels of cholesterol and triglycerides were measured using Infinity Cholesterol and Infinity Triglycerides (Triolab AB, Gothenburg, Sweden) with Multiconstituent Calibrator 1E65-04 (Abbott, Solna, Sweden) used as reference.

### Statistical Analysis

The non-parametric Mann-Whitney U-test was used to investigate differences between hSAA1 mice and their wt littermates. Spearman rank correlation test was used to assess correlation between adipose tissue hSAA1 gene expression and plasma levels of hSAA. Possible differences in growth rate were analyzed with repeated measures analysis of variance (ANOVA). All statistical analyses were performed using PASW 19.0 (Chicago, IL). Data are presented as mean ± SEM. A p-value of less than 0.05 was considered significant.

## Results

### Animal Growth Curves

Male hSAA1 mice (n = 33) displayed no significant difference in weight compared to wt controls (n = 23) at 11 weeks of age (28.4±0.3 g and 28.8±0.3 g, respectively) or at 35 weeks of age (37.2±0.6 g and 38.2±0.8 g, respectively). In line with previous results [30], growth curves for male hSAA1 mice and their wild type littermates were almost identical (data not shown).

### SAA Gene Expression in Adipose Tissue and Plasma Levels of SAA

The human SAA1/2 was expressed in gonadal adipose tissue in hSAA1 mice and undetectable in wt mice. Plasma levels of human SAA were in the same range as previously reported for hSAA1 mice fed normal chow [29] and correlated with mRNA levels of hSAA1 in gonadal adipose tissue. As shown previously in hSAA1 mice fed a high fat diet [29], [30], mRNA levels of mouse Saa3 in gonadal adipose tissue displayed a trend towards down-regulation in hSAA1 mice compared to wt mice. The same pattern was also seen for plasma levels of mouse SAA.

### Plasma Levels of Cholesterol and Triglycerides

hSAA1 mice and their wt controls displayed similar plasma levels of cholesterol (13.2±0.6 mmol/l and 14.0±0.7 mmol/l, respectively) and triglycerides (1.5±0.1 mmol/l and 1.6±0.1 mmol/l, respectively).

### Quantification of Atherosclerotic Lesions

**Figure 1:**
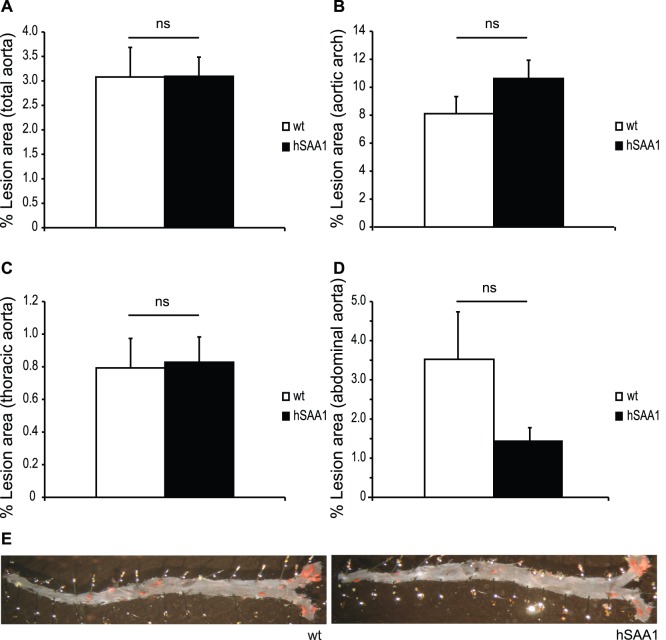
Quantification of atherosclerotic lesion area in *en face* prepared aortas from male hSAA1 (n = 33) and wt (n = 23) mice on ApoE-deficient background. Lesion area with positive Sudan IV staining is expressed as the percentage of total area in (A) total aorta, (B) aortic arch, (C) thoracic aorta and (D) abdominal aorta. Data are presented as mean ± SEM. ns = non significant with Mann-Whitney U-test. (E) Photographs illustrating atherosclerotic lesions in wt and hSAA1 mice.

Quantitative computer-assisted image analysis of aortas prepared *en face* revealed no significant difference in atherosclerotic lesion area in hSAA1 mice compared to wt mice (Figure 1). The mean atherosclerotic lesion area was similar in the thoracic aorta for hSAA1 and wt mice (0.83±0.15% and 0.79±0.18%, respectively, p = 0.835). The hSAA1 mice displayed a trend towards increased atherosclerotic lesion area in the aortic arch compared to wt mice (10.62±1.31% and 8.11±1.22%, respectively, p = 0.254). The opposite trend was seen in the abdominal aorta where hSAA mice displayed decreased atherosclerotic lesion area compared to wt mice (1.43±0.34% and 3.52±1.21%, respectively, p = 0.720). However, none of these differences were statistically significant and the mean atherosclerotic lesion area in the whole aorta was similar in hSAA1 mice and wt mice (3.09±0.39% and 3.08±0.60%, respectively, p = 0.306).

## Discussion

We show in this study that chronic moderately elevated levels of human SAA derived from adipose tissue does not affect atherosclerotic lesion area in hSAA1^+/−/^ApoE^−/−^ mice. Data from aortas analyzed *en face* demonstrate that hSAA1 mice on an ApoE-deficient background develop atherosclerotic lesions to the same extent as their wt littermates in all sections of the aorta. Several studies have reported links between elevated levels of circulating SAA and atherosclerotic disease [2]–[5]. In addition, SAA mRNA and SAA protein are present in atherosclerotic lesions [6]–[8]. However, whether SAA plays a causal role in atherosclerosis is unknown. Reports have suggested that SAA has pro-atherogenic effects [8], [15], [19]–[21], [31]–[33]. Overexpression of human SAA1 in mice and moderate inflammation impair HDL-mediated reverse cholesterol transport [15], [34]. Previous studies also suggest that SAA promotes lipoprotein retention in the vessel wall by increasing proteoglycan synthesis in smooth muscle cells and by facilitating the binding of HDL to proteoglycans [8], [19], [20], [24]. However, several reports also suggest anti-atherogenic functions of SAA [18], [22], [25], [26], [35], [36]. The SAA-induced impairment of reverse cholesterol transport has been questioned and some data suggest that SAA promotes cholesterol efflux from macrophages, thereby having possible anti-atherogenic effects [18], [22], [37], [38].

In this study we have analyzed whether a chronic increase in circulating hSAA derived from adipose tissue influences the development of atherosclerosis in ApoE-deficient mice. We show that the hSAA1 mice display atherosclerotic lesion areas to the same extent as their wt littermates. Our results are in line with a previous study where mice deficient in endogenous SAA develop atherosclerosis to the same extent as their wt littermates [39]. However, one study has shown that increased levels of mouse SAA1, induced by lentiviral over-expression, lead to increased atherogenesis [28]. In contrast, administration of mouse SAA2 peptides to mice prevents and reverses atherosclerotic lesion development [26]. The differences in results between *in vivo* models may be due to the type of SAA investigated, the model used or the source of SAA over-expression. Our hSAA1^−/+^ mouse model is designed to mimic the human state in non-acute phase where SAA1 is produced in the adipose tissue [29]. The hSAA1^+/−^ mice chronically display moderately elevated levels of human SAA [29], and the model is suitable for investigating long-term effects of the chronically elevated SAA levels often seen in patients with obesity and/or atherosclerosis. We have previously shown that adipose tissue-derived human SAA does not influence the development of insulin resistance or adipose tissue inflammation in hSAA1 mice [30] and in this report we show that hSAA1 mice on an ApoE-deficient background develop atherosclerotic lesions to the same extent as their wt littermates in all sections of the aorta.

In conclusion, we here show that chronically moderately elevated levels of human SAA derived from adipose tissue does not affect atherosclerotic lesion area in hSAA1^+/−/^ApoE^−/−^ mice. Our data suggest that human serum amyloid A originating from the adipose tissue is not a mediator of atherosclerotic disease in mice.
